# Short-term effects on heart rate variability of occipito-mastoid suture normalization in healthy subjects

**DOI:** 10.3389/fnins.2023.1271461

**Published:** 2023-09-25

**Authors:** Cyril Besson, Thierry Mur, Charles Benaim, Laurent Schmitt, Vincent Gremeaux

**Affiliations:** ^1^Department of Sports Medicine, Swiss Olympic Medical Center, Lausanne University Hospital, Lausanne, Switzerland; ^2^Institute of Sports Sciences, University of Lausanne, Lausanne, Switzerland; ^3^Department of Physiotherapy, Aquamed Center, Montreux, Switzerland; ^4^Department of Physical Medicine and Rehabilitation, Lausanne University Hospital, Lausanne, Switzerland; ^5^National School of Mountain Sports/National Ski-Nordic Centre, Premanon, France

**Keywords:** osteopathy manual therapy, vagal modulation, cardiac autonomic control, skull, SHAM

## Abstract

Occipito-mastoid structure normalization (OMSN) is an osteopathic manipulative treatment aimed at reducing tension around the jugular foramen, where cranial nerves IX, X, and XI exit the skull. The purpose of this study was to observe how heart rate variability (HRV), a marker of autonomic cardiac regulation, was modulated after an OMSN vs. a sham technique (SHAM). Pre- and post-intervention HRV was analyzed in two randomly chosen groups of 15 participants (OMSN vs. SHAM group). HRV was collected in the supine position 5 min before and 5 min after a 10-min application of either OMSN or SHAM. The time and group effect was analyzed using a two-way ANOVA. Independently from group intervention, a significant time effect induced increased HRV. No group effect differences were observed. Multiple comparisons for time and group interaction showed that the root mean square of successive differences (RMSSD), a vagally mediated HRV variable, increased to a greater extent for the OMSN group (*p* = 0.03) than for the SHAM group. However, both OMSN and SHAM techniques had a significant effect on HRV. Compared to a SHAM technique, OMSN had a significant effect on HRV vagally related metric RMSSD in the short term. We conclude that 10 min of OMSN may be used to induce a short-term influence on parasympathetic autonomic nervous system modulations.

## 1. Introduction

Osteopathy promotes health *via* a patient-centered holistic approach. The term osteopathic manipulative treatment (OMT) encompasses non-invasive forms of manual therapy using palpation and manual techniques to influence body-tissue interconnection and to maintain or restore health (Carnevali et al., 2020). Among the assumed outcomes, OMT is believed to influence the autonomic nervous system (ANS), which in turn is essential to general health (Rees, 2014; Rechberger et al., 2019; Carnevali et al., 2020). The ANS regulates physiological functions, maintaining body homeostasis according to internal and external variations. It is composed of two main branches: (1) the parasympathetic system (PS) has a craniosacral anatomical organization, which predominates in periods of calm, in particular, to regenerate the body and (2) the orthosympathetic system (OS) has a predominant dorsolumbar organization, which produces “fight or flight” reactions in the face of an impending threat (McCorry, 2007; Wehrwein et al., 2016). An imbalance in the ANS is called dysautonomia and has numerous causes (Reichgott, 1990). They can be functional, for instance, a structural alteration of functional ANS anatomy (e.g., compression or inflammation) and can be affected by different stressful stimuli (Grossman and Taylor, 2007; Porges, 2009; Thayer and Lane, 2009). Osteopathy is among the different treatments available for dysautonomia (Rechberger et al., 2019). A meta-analysis that found 23 studies on osteopathy efficacy on ANS, which incorporated three main techniques [high-velocity low-amplitude techniques (HVLAT); cranial OMT techniques; mobilization techniques in the thoracic and cervical spine], highlighted those as follows: (1) Significant changes in ANS activity may happen with HVLAT, (2) no conclusion could be drawn from studies on cranial osteopathy due to lack of methodological quality, (3) significant changes in ANS activity may happen in suboccipital region treatments, (4) no conclusion could be drawn from studies on cervical and thoracic mobilization, (5) whether activations happen in the sympathetic or parasympathetic part of ANS is unclear, and (6) thorough methodological research must be conducted (Rechberger et al., 2019). The authors concluded that OMT can positively influence ANS. However, they highlighted that different OMTs were performed using different techniques, regions, and ways of evaluating the ANS activity.

Heart rate variability (HRV) is used to interpret ANS cardiac control with the advantage of being non-invasive and easy to collect (Grossman and Taylor, 2007; Porges, 2009; Thayer and Lane, 2009). It is employed as a quantitative and qualitative stress assessment, especially by measuring cardiac vagal tone activity (Malik, 1996; Laborde et al., 2017). Literature has recently focused on the osteopathic effects on the ANS using different techniques and treatment locations. However, an improved organization of research protocols is required (Steel et al., 2017; Rechberger et al., 2019; Carnevali et al., 2020). In a recent perspective article, Carnevali et al. considered that heart rate variability (HRV) analysis can be used to evaluate the effectiveness of OMT. Indeed, some HRV variables may reliably be interpreted as reflecting cardiac vagal tone using measures such as the root mean square of successive beat-to-beat interval differences (RMSSD) or the high-frequency (HF) power spectrum (Laborde et al., 2017; Shaffer and Ginsberg, 2017). Among studies cited by Rechberger's meta-analysis, Giles et al. showed an increase in the HF power spectrum after suboccipital decompression (Giles et al., 2013). Another study showed a decrease in sympathetic activity after a suboccipital manipulation (Purdy et al., 1996).

Cranial nerves IX, X, and XI [glossopharyngeal, vagus, and accessory (or spinal accessory)] pass through the jugular foramen between the occiput and the temporal bone of the skull. Dysfunction of the jugular foramen may cause dysautonomia, and thus, OMT techniques may be adopted in certain cases. As the vagus nerve is one of the main parasympathetic nerves (Gerritsen and Band, 2018) and its activity is closely related to homeostasis, treatments targeting the jugular foramen area may have a certain relevance in ANS balance. Sergueef described the occipito-mastoid suture normalization (OMSN) to be effective in normalizing tensions between the occiput and the temporal bone (Sergueef, 2018). Its purpose is to achieve a state of equilibrium of tensions in the treated area, favorable to mechanical, circulatory, and neurological harmonization. However, there is a scarcity of related research in this area and even if this technique may be used for treating an imbalance, no current research exists on possible effects in healthy participants. To the best of our knowledge, no study has investigated the objective non-invasive measurement of stress *via* HRV after an OMSN OMT neither in patients nor in healthy participants. This study aimed to describe whether a cranial technique of normalization of the occipito-mastoid suture may influence cardiac vagal activity HRV-related indexes in a priori asymptomatic participants. We hypothesized that parasympathetic nervous system (PNS) HRV markers will increase to a greater extent compared to a sham technique (SHAM) following an OMSN OMT session in healthy subjects.

## 2. Materials and methods

### 2.1. Participants and study design

The criteria for inclusion were to be generally healthy and be aged between 18 and 75 years old. The exclusion criteria included primary or secondary dysautonomia, head trauma-induced dysautonomia, and treatment influencing cardiorespiratory function. Thirty-four people participated voluntarily in the study. which was approved by the Canton de Vaud ethics committee (#2020-02867) in accordance with the ethical standards of the Helsinki Declaration. All participants signed an informed consent form.

A prospective, interventional study, single-blinded, randomized control trial design was used to compare HRV pre- and post-OMSN or SHAM techniques. Participants were included for a single visit to the physiotherapy center Aquamed Malley in Lausanne. They were equipped with a heart rate monitor and were then randomly assigned to either the OMSN or SHAM group (single blinded). Participants were installed in a supine position on a physical therapy table, with their eyes closed, and were asked not to think about anything in particular. After ensuring that there was no discomfort (e.g., excessive belt tension or feeling of cold), participants remained in that position for 3 min before R-R intervals were recorded for a period of 5 min. Once this first R-R interval measurement period was over, the investigator applied either OMSN or SHAM treatments for 10 min. R-R intervals were then collected again during a 5-min period following the intervention.

### 2.2. Heart rate variability

To minimize confounding factors on HRV, participants were asked to attend the laboratory under standardized conditions: no training or intense physical activity in the 24 h preceding the visit, no muscle soreness, fasted or to have finished their last meal at least 3 h before the beginning of the protocol, no alcohol, tea or caffeine ingestion or smoking during the last 12 h. They were asked not to attend if they showed signs of illness. It is known that data obtained for a short-term intra-individual HRV analysis remain interpretable as being less impacted by external factors (Quintana and Heathers, 2014). However, we standardized experimental conditions according to the following criteria: indirect and equal intensity lighting, constant and comfortable temperature, no particular noise or smell, communication limited to set-up, and protocol presentation instructions. The participants were asked not to move or engage in conversation unless they needed to communicate important information or experienced discomfort.

Participants were equipped with a Polar H10 heart rate monitor paired with a Polar V800 watch (Polar Electro Oy, Kempele, Finland) to collect R-R intervals. R-R intervals were extracted from the Polar online platform and visually inspected for artifacts and ectopic beats, which were automatically and manually corrected using Kubios Premium (Kubios, Finland) (Tarvainen et al., 2014). An experienced exercise physiologist blinded to which group participants belonged to performed the analysis. The final 4 min of each 5 min samples were analyzed as it allowed a reliable frequency-domain analysis (Bourdillon et al., 2017). Time- and frequency-domain and non-linear variables were kept for analysis (Shaffer and Ginsberg, 2017), with particular attention being paid to vagally mediated variables [e.g., RMSSD, HR, HF, HF relative to HR (HF/HR), (LF+HF)/HR, and detrended fluctuation analysis alpha 1 (DFA1)].

### 2.3. Occipito-mastoid suture normalization

**Figure 1:**
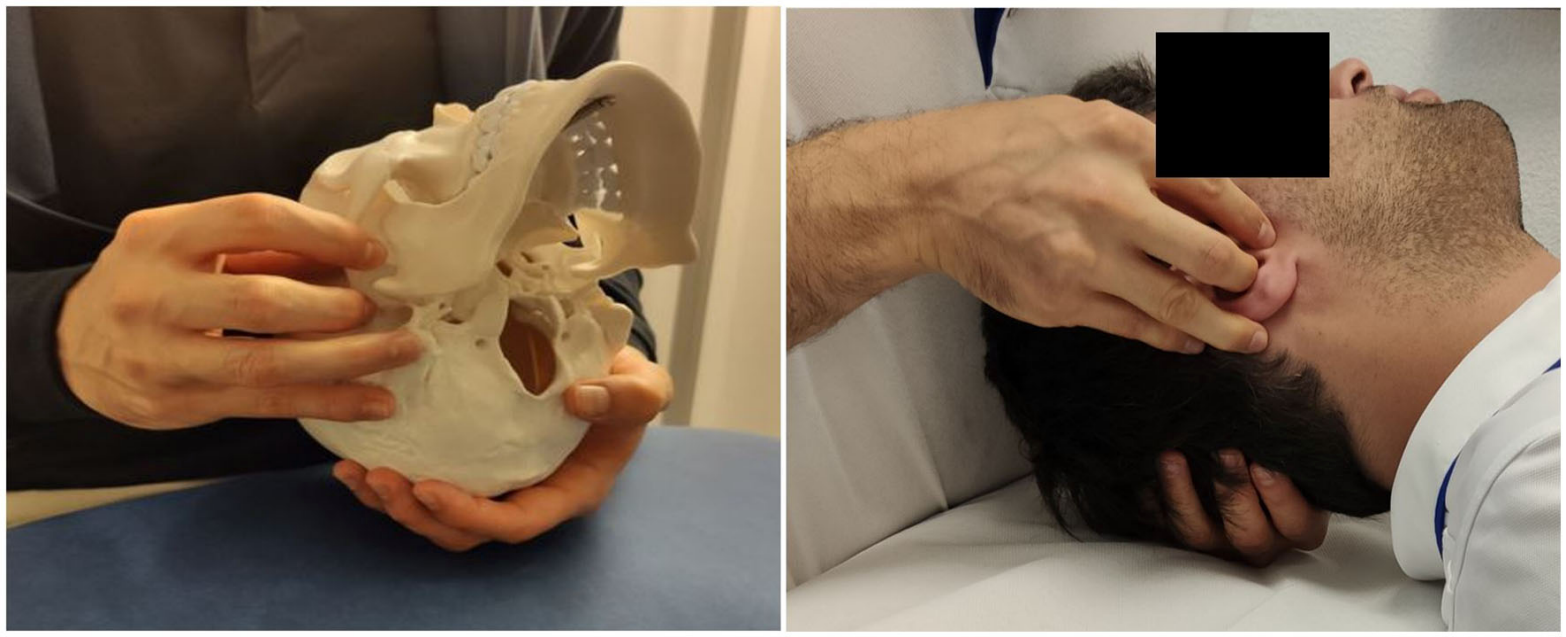
Manual grasp for the right OMSN on the skeleton (for illustration) and with a participant.

The OMSN technique (illustrated in Figure 1) is a passive and smooth osteopathic technique, regularly applied in general practice. The participant is in the dorsal decubitus position with the practitioner by his head. The investigator places his contralateral hand opposite the treated side transversally under the occiput with the pulp of the index, middle, and ring fingers under the occipital scale, medial to the occipito-mastoid structure. The homolateral hand is placed on the temporal bone with a five-finger grip: the thumb and index finger above and below the zygomatic process, respectively, the medius at the level of the external acoustic pore, the ring finger on the tip of the mastoid process, and the little finger on the mastoid portion. Functional treatment begins with feeling and then continues with tissue guidance in the direction of dysfunction across the three spatial planes where improved movement is sensed. The practitioner gradually facilitates these movements until a noticeable relaxation occurs, indicating normalization. The goal is to establish a state of balanced tension in the treated area, promoting alignment among mechanical, circulatory, and neurological mechanisms. Using a perceptive and light touch, no additional pressure other than simply accompanying the tissues in the direction of their ease is applied. In the present study, the technique was applied for 10 min, 5 min for each jugular foramen. For the SHAM group, the investigator just positioned the fingers as for OMSN, but with no intention of feeling nor treating.

### 2.4. Statistical analyses

The data are expressed as mean ± standard deviation (SD). Differences between groups in age, weight, height, and body mass index (BMI) were tested with unpaired *t*-tests. A two-way repeated measures ANOVA [time (pre- vs. post-) × group (OMSN vs. SHAM)] was performed to assess for an intervention effect. This analysis allowed the identification of differences between pre- and post-intervention (time effect) and within the intervention (group effect). All data passed Mauchly's sphericity test. When repeated measures ANOVA revealed a significant main effect (time or group) or interaction effect, multiple comparisons were performed to test the significance of the differences using Bonferroni adjustments. All statistical analyses were performed with IBM SPSS version 28 software (IBM Corp., Armonk, NY, USA). The level of significance was set to be at a *p* ≤ 0.05.

## 3. Results

In total, 34 participants were randomly assigned to either the OMSN or SHAM groups. Once a group attained 17 participants, the last participants entering the study were attributed to the other group. The first two participants were excluded because the technique applied was incorrect. Two other participants were excluded from the analysis: one because of non-compliance with the protocol instructions (open eyes, repeated movements) and one who showed signs of arrhythmia and was referred for a cardiac check-up. Each excluded participant was replaced by a participant with the same intervention. A final number of 21 women and 9 men were included in the analysis. Groups were considered as homogenous as no difference in gender distribution, age, weight, or height was observed (see Table 1).

**Table 1:** Gender repartition, age, weight, and height of participants.

	**OMSN**	**SHAM**	***P*-value**
M/F	4/11	5/10	
Age (year)	38 ± 16.5	37.1 ± 13.7	0.877
Weight (kg)	68.9 ± 12.3	69.5 ± 7.4	0.858
Height (cm)	168.3 ± 11.5	169.1 ± 7.5	0.822
BMI (kg/m^2^)	24.3 ± 3.3	24.5 ± 3.4	0.884

Data are mean ± SD. OMSN: occipito-mastoidian suture normalization; M, male; F, female.

Table 2 presents pre- and post-intervention values and *p*-values for each variable and condition.

**Table 2:** Comparisons of time- and frequency-domain and non-linear HRV variables pre- and post-OMSN and SHAM.

	**OMSN**		**SHAM**		**Two-way ANOVA** ***p*****-value**		
	**PRE**	**POST**	**PRE**	**POST**	**Time effect**	**Group effect**	**Time** ^*^ **group interaction effect**
SDNN (ms)	40.6 ± 21.1	53.5 ± 26.6	50.8 ± 30.6	57.2 ± 28.2	**0.002**	0.461	0.26
HR (bpm)	73.2 ± 10.7	68.6 ± 8.5	67 ± 10.7	64.6 ± 9.6	**< 0.001**	0.164	0.108
RMSSD (ms)	**29.3** **±21**	**37.4** ±21.7^*^	42.6 ± 39.2	45.4 ± 35.6	**< 0.001**	0.345	**0.03**
PNN50 (%)	8.8 ± 12.2	15.4 ± 16.4	19.7 ± 25.1	22.9 ± 25.1	**< 0.001**	0.222	0.152
VLF (ms^2^)	631.3 ± 551	1247.5 ± 1532.3	967.2 ± 996.4	1770.4 ± 2329	0.056	0.314	0.795
LF (ms^2^)	758.7 ± 1220.2	1317 ± 1876.7	831.2 ± 1175.4	1020.3 ± 971.7	**0.041**	0.81	0.299
HF (ms^2^)	540.9 ± 750.3	683.6 ± 987.3	1032.6 ± 1442.5	1075.3 ± 1359.9	0.15	0.305	0.432
LF/HF	2.6 ± 3.1	2.9 ± 3.5	2.4 ± 2.7	2.2 ± 2.1	0.646	0.653	0.351
LF+HF (ms^2^)	1299.6 ± 1864.8	2000.6 ± 2781.8	1863.7 ± 2243.6	2095.6 ± 1932.8	**0.022**	0.681	0.234
TP (ms^2^)	1930.9 ± 2251.3	3248.1 ± 4244.3	2830.9 ± 3164.2	3866 ± 3831.4	**0.02**	0.521	0.77
LF (nu)	60.8 ± 17.3	64.1 ± 18	55.6 ± 24	57 ± 22.5	0.455	0.379	0.761
HF (nu)	39.2 ± 17.3	35.9 ± 18	44.4 ± 24	43 ± 22.5	0.455	0.379	0.761
HF/HR	8.1 ± 11.9	10.7 ± 16.5	18.2 ± 27.2	19.3 ± 26.4	0.101	0.239	0.496
(LF+HF)/HR	19.5 ± 30.3	31.3 ± 47.2	32.8 ± 44.9	37 ± 40.3	**0.027**	0.523	0.523
Poincare plot, SD1 (ms)	20.8 ± 14.9	26.5 ± 15.4^*^	30.2 ± 27.8	32.2 ± 25.2	**< 0.001**	0.345	**0.03**
Poincare plot, SD2 (ms)	53.1 ± 26.5	70.6 ± 34.7	64.4 ± 34.7	73 ± 34	**0.006**	0.544	0.32
Poincare plot, SD2/SD1	2.95 ± 0.95	2.92 ± 0.85	3.01 ± 1.46	2.82 ± 1.08	0.516	0.959	0.632
ApEn	1.06 ± 0.09	1.02 ± 0.09	0.99 ± 0.1	0.98 ± 0.09	0.198	0.086	0.375
SampEn	1.51 ± 0.24	1.47 ± 0.21	1.51 ± 0.36	1.53 ± 0.4	0.813	0.764	0.54
DFA, alpha 1	1.19 ± 0.24	1.23 ± 0.26	1.07 ± 0.3	1.08 ± 0.3	0.396	0.175	0.573
DFA, alpha 2	0.88 ± 0.25	0.82 ± 0.25	0.93 ± 0.2	0.86 ± 0.2	0.218	0.519	0.889
Correl dimension, D2	1.84 ± 1.51	2.4 ± 1.45	2.16 ± 1.6	2.12 ± 1.49	0.332	0.965	0.272

Data are mean ± SD.

OMSN, occipito-mastoidian suture normalization; SDNN, standard error of normal-to-normal interval; HR, heart rate; RMSSD, root mean square of successive difference; PNN50, percentage of successive R-R intervals that differ by >50 ms; VLF, very low frequencies; LF, low frequencies; HF, high frequencies; LF/HF, ratio LF in function of HF; TP, total power; nu, normalized units; ApEn, approximate entropy; SampEn, sample entropy; DFA, detrended fluctuation analysis; Corell, correlation.

* Variables statistically different with *post-hoc* analysis. Bold font indicates statistical significance.

Independently from group intervention, a significant time effect was observed for SDNN, RMSSD, PNN50, LF, LF+HF, TP, (LF+HF)/HR, SD1, and SD2, which all increased, whereas HR decreased. No time effect was observed in HF. Group effect analysis showed no differences between interventions. When analyzing multiple comparisons for time and group interaction, RMSSD increased to a greater extent for the OMSN group (*p* = 0.03).

## 4. Discussion

The objective of this study was to observe how HRV is modulated after a particular OMT. We showed that, after OMSN, RMSSD increased to a greater extent than a SHAM condition. HRV increased in both groups with several vagally mediated variables increasing independently from group intervention. This response was expected because constant adaptation to position occurs *via* the ANS and cardiovascular centers. Proprioceptors, chemoreceptors, and baroreceptors influence HR adjustment *via* the ANS, which is likely to decrease cardiac output (Shaffer et al., 2014). Given that manual contact happened also in SHAM, even without the intention of feeling and treating, an inherent effect on the patient's physiology may have occurred independently of a placebo effect, which itself may also have had an effect (Meissner, 2011; Benedetti, 2013; Cerritelli et al., 2016). No changes in vagally mediated HRV frequency-domain variable HF were found. The intention and application of the OMSN technique increased vagal tone (as evidenced by RMSSD increase), at least directly following a 10 min application.

OMTs allow a relaxation of myotendinous, aponeurotic, and even bony tissues for the skull according to Sutherland's clinical observations and, consequently, improved local vascular-nerve functioning (Sutherland, 2000; Sergueef, 2018). However, to date, those clinical observations remain unproven as do assumptions about cranial osteopathy. Some upper cervical OMT studies have shown influences on the PNS. In his thesis, Vaudron reported that among several cranial, facial, and osteoarticular techniques, the OMT technique applied to the occipito-mastoid suture had the greatest impact on the ANS, evaluated using a sitting HRV method (Vaudron, 2018). Furthermore, Rechberger et al., in their systematic review of 23 studies on the influence of osteopathy on the ANS, highlighted that despite a low number of participants, most selected studies showed an effect on the ANS (Rechberger et al., 2019), especially HVLAT and treatments of the suboccipital region. However, mechanisms underlying the increase in ANS activity remain unclear and are surprisingly not discussed in Rechberger's study. Importantly, they stated that no conclusion can be drawn as to whether OMT has a bigger impact on SNS or PNS activity. Our data suggest an increase in variables linked to both system activities (e.g., LF + HF and TP), with signs of an assumed parasympathetic increase, confirmed through the observed higher RMSSD following the OMSN technique. Carnevali et al. have proposed that the mechanisms of OMT influencing ANS are currently unclear (Carnevali et al., 2020), but they proposed two hypotheses: (1) musculoskeletal alterations may increase pro-inflammatory mediators, and thus sympathetic activity. Their corrections may then reduce sympathetic responses and improve HRV. Corrections around the vagus nerve may improve anti-inflammatory responses. However, in our study, it would seem difficult to imagine such a process happening in the minutes after the intervention. (2) Given that increased HRV after OMTs is also described in healthy individuals, other mechanisms should be involved. For example, there is evidence that the activation of low-threshold C mechanoreceptors and their connections to the insular cortex may positively influence the ANS (Carnevali et al., 2020). This may have likely been the case in our study.

Studies investigating OMT and HRV differed in the type of OMT performed, the population studied, and the HRV method used. For example, Henley et al. performed a cervical myofascial OMT, a touch-only sham OMT, and no-touch control on 17 healthy subjects while at a 50-degree head-up tilt (Henley et al., 2008). They showed an increase in HF when OMT was performed during the tilt, attesting to an acute effect on PNS activity during treatment. However, no significant effect was reported between pre- and post-treatment comparisons. They stated that head tilting induced a greater sympathetic tone, but it has also been shown that head-down neck flexion induces a parasympathetic withdrawal (Lee et al., 2001). The authors discuss this mechanism with the role of neck afferences and influences of neck flexion activating autonomic reflexes and vascular control *via* the stimulation of otolith organs.

Several studies have shown a positive influence of OMT on HF vs. a sham technique, supporting an increase in the parasympathetic tone of such an intervention. Giles et al. showed a significant increase in HF after a 15-min OMT including soft tissue manipulation in the cervical region and suboccipital decompression (Giles et al., 2013). Using an 8-min cranial OMT (augmentation + suppression) technique, Shi et al. evidenced a decreased sympathetic and an increased parasympathetic modulation post-treatment in 21 healthy participants (Shi et al., 2011). Both studies measured HRV in the supine position. In a randomized control trial (RCT) including 66 participants, Rufini et al. showed a positive effect on HRV vagally related variables of OMT (Ruffini et al., 2015); indeed, Cerritelli et al. arrived at similar conclusions in a cohort of 37 healthy participants. Ruffini et al. presented a non-linear analysis and showed that OMT significantly decreased DFA alpha 1 compared to a control group (p < 0.001) and with a trend compared to sham therapy (p = 0.09). However, in both studies, OMT intervention was based on the patient's request even if they were included as healthy (Cerritelli et al., 2020). In comparison to the above studies, we did not show any significant differences in vagally mediated HRV variables, except for RMSSD. Standard deviations in the frequency domain showed extreme inter-individual variations which make it difficult to conclude that there was an effect on HF. In our opinion, the best way to question parasympathetic tone *via* HRV is to analyze different vagally related HRV variables in parallel. Despite finding that only one significant variable increased after OMSN, our results are in line with different studies on OMT which seem to validate parasympathetic-related HRV increased after treatment (Shi et al., 2011; Giles et al., 2013; Ruffini et al., 2015; Cerritelli et al., 2020). Several studies show that OMT has an autonomic effect measured by HRV in healthy subjects, showing mainly an increase in vagal modulation and being independent of sex.

### 4.1. Limitations and recommendations

During our protocol, the application of the technique lasted only 10 min, which is relatively short if we consider the handling and feeling for each jugular foramen takes at least 1 min. However, given the short protocol duration, our results are promising as there is the possibility of an even greater effect of OMSN on ANS, in the event of a longer application of the technique. We may also question the post-treatment duration effect. Indeed, it is difficult to know if the effect observed lasts for a significant period of time for the participant, and research on chronic effects should be conducted to investigate this. Moreover, as stated by Carnevali et al., the mechanisms behind increased vagal tone after OMT need to be clarified (Carnevali et al., 2020). Indeed, despite performing a maneuver aiming directly to mobilize the bone around the jugular foramen in our study, we could not exclude influences, even minor, of OMSN on mechanical, circulatory, and neurological mechanisms from other structures around the treated area on ANS modulation. For instance, nerve activity from the carotid canal (carotid sinus nerve or Hering's nerve), which is a small branch of the cranial nerve IX (Glossopharyngeal) that innervates the carotid sinus, and carotid body, may also influence ANS activity through short-term modification of blood pressure regulation. From a sports medicine perspective, as vagal tone is reduced post-moderately to high-intensity exercise, using OMSN may for instance help to improve recovery in athletes, but this needs to be confirmed through scientific evidence. Finally, considering our results on healthy subjects, investigations on patients (e.g., dysautonomia or hypertensive patients) should be considered.

Only one investigator performed the techniques on all the participants which allowed for standardized interventions. Thus, the conclusions of this study therefore concern only one practitioner. Although the technique used is not particularly complex to perform, an inter-practitioner application (involving different operators) would increase the external validity of this study. A larger sample size would also improve the generalizability of the technique and results.

The transposition of RCT methodology to the field of osteopathy cannot be complete due to the nature of the care tested. Indeed, the implementation of a double-blind design presupposes that an osteopath can perform a manual treatment without knowing if it is a real or a fake treatment, which is impossible. In this study, the operator learns the technique (OMSN or SHAM) to be applied as late as possible once the patient is settled with eyes closed and the cessation of non-essential communication is recorded. These measures were intended to minimize the single-blind bias inherent in manual therapy studies. These methodological difficulties, and even drawbacks, weaken the internal validity of this study. Moreover, as Cerritelli et al. pointed out in a systematic review of the methodology of osteopathic RCT-type studies, there are still no “guidelines” on the procedure for setting up sham treatment or placebo groups (Cerritelli et al., 2016). The methodology upon which we relied is therefore found in the majority of manual therapy studies. However, the heterogeneity of the protocols described in the literature weakens the reproducibility of the results, which makes it difficult to triangulate the conclusions between studies.

## 5. Conclusion

This study aimed to measure the impact of an OMSN on the ANS through the instrumental measurement of HRV. We were able to describe evidence of increased vagal tone post-intervention versus a control group. The various methodological biases, mainly related to the sample size and the limitations of the RCTs concerning manual care, put these results into perspective. However, the observations made are consistent with other studies concerning the location of application of the technique (suboccipital region) and the desired effect (increase in vagal tone). Positively influencing vagal tone with OMT has drastic practical implications (Carnevali et al., 2020). Other conditions such as dysautonomia, hypertensive patients, or stress management may benefit from this technique. Further studies may in future confirm the validity of these results.

## Data availability statement

The raw data supporting the conclusions of this article will be made available by the authors, without undue reservation.

## Ethics statement

The study involving humans were approved by the Commission cantonale d'éthique de la recherche sur l'être humain (CER-VD). The study were conducted in accordance with the local legislation and institutional requirements. The participants provided their written informed consent to participate in this study.

## Author contributions

CBes: Conceptualization, Formal analysis, Software, Writing—original draft, Writing—review and editing. TM: Conceptualization, Data curation, Investigation, Writing—original draft, Writing—review and editing. CBen: Methodology, Software, Writing—review and editing. LS: Formal analysis, Writing—review and editing. VG: Supervision, Writing—review and editing.
